# Assessing the relationship between incisor wear, age, and body condition in Dohne Merino ewes (*Ovis aries*)

**DOI:** 10.1017/awf.2025.10027

**Published:** 2025-07-30

**Authors:** Alexandra Sophie Holt, Fritha M. Langford

**Affiliations:** 1 School of Natural and Environmental Science, Newcastle University, Newcastle-upon-Tyne, UK; 2 The University of Edinburgh Royal Dick School of Veterinary Studies, Roslin, Midlothian, UK

**Keywords:** Animal welfare, body condition score, dental disorder, incisor, sheep, wear

## Abstract

Sheep (*Ovis aries*) are stoic, prey animals that have evolved to mask signs of pain and vulnerability, making behavioural indicators of poor welfare difficult to detect. Body condition scoring (BCS) remains one of the most practical, animal-based indicators of chronic undernutrition and compromised welfare in grazing ruminants. Disruption of the incisor apparatus due to dental disorders or tooth loss can impair grazing efficiency, resulting in reduced nutritional intake and contributing to poor body condition. Despite this, there has been little research into the prevalence or welfare impact of dental disorders in sheep. This preliminary study aimed to assess the prevalence of incisor wear in extensively grazed Dohne Merino ewes, examine its distribution across age groups, and evaluate associations with bodyweight and BCS. A total of 818 ewes aged 2 to 10 years were evaluated during routine husbandry. Incisor wear was scored based on dentine exposure using a 0–3 ordinal scale. BCS was determined through hands-on palpation, and liveweight was recorded. Wear affecting more than one-third of tooth enamel was present in at least one incisor in 99% of ewes over five years of age. Greater incisor wear was significantly associated with lower bodyweight and BCS in ewes over two years, irrespective of age. These findings underscore the potential role of incisor wear as a contributing factor to nutritional compromise and welfare risk in older sheep. Monitoring incisor health may facilitate more accurate welfare assessments and enhance management strategies in extensive systems.

## Introduction

The behavioural stoicism of sheep (*Ovis aries*), shaped by their evolutionary role as prey animals, makes recognising pain and poor welfare particularly challenging. Observable indicators are often minimal or absent, limiting the effectiveness of behavioural assessments in welfare monitoring. In extensive grazing systems, body condition score (BCS) is widely used as an indirect yet valuable animal-based indicator of welfare, reflecting longer-term changes in nutrition and health. Dental disorders, particularly the breakdown of the incisor apparatus through wear, disease, or tooth loss, can hinder effective grazing and access to feed. This, in turn, can lead to undernutrition, which is detectable through a decline in BCS. Investigating the relationship between incisor health and body condition provides important insight into the welfare implications of dental disease in sheep.

Incisor disorders in sheep are potentially painful conditions that lead to the loss of incisor function, resulting in reduced feed intake, bodyweight, BCS, and milk and fibre production (Sykes *et al.* 1974; McGregor & Butler 2015; Dove *et al.* 2018). The size and shape of the incisor arcade directly determine bite dimensions and grazing efficiency; dental disorders can alter or degrade the incisor apparatus, impacting the animal’s ability to graze effectively (Hongo *et al.* 2004). The occurrence of dental disorders in sheep has traditionally been considered the result of periodontitis or wear; periodontitis in sheep is also called broken mouth periodontitis (BMP), an inflammatory disease that progresses to the destruction of the periodontal ligament, ultimately causing tooth loss (Riggio *et al.* 2013). In contrast, wear is described as a non-carious loss of tooth tissue, a normal physical process that occurs over time (Bishop *et al.* 1997). Periodontitis affects the periodontal ligament, critical in anchoring the incisors and providing viscoelasticity to tolerate grazing pressures, allowing up to 2 mm of movement (Cutress 1972; Moxham *et al.* 1987). However, these same features make the sheep incisor prone to loss following wear, disease and damage (Spence & Aitchison 1986). The periodontal ligament also contains nerves capable of detecting temperature, pain and pressure (Jansen *et al.* 2022). It is essential to distinguish between wear and periodontitis, as both conditions can independently lead to tooth loss, albeit via different mechanisms.

The term ‘excessive wear’ has been used in previous sheep dental studies to describe cases of significant dental tissue loss, often implying that only the most severe instances are of concern (Bruere *et al.* 1979; Thurley 1984; Laws *et al.* 1988; Orr & Chalmers 1988; Frisken *et al.* 1989; West 2002; Ridler & West 2007; Borsanelli *et al.* 2021). However, it is now recognised that the term ‘excessive wear’ may not accurately describe the multifactorial nature of tooth wear or its progression. Tooth wear is better understood as a spectrum of conditions influenced by various factors such as grazing behaviour, diet, and environmental conditions. By focusing exclusively on severe cases, the literature may have overlooked the broader implications of tooth wear on function, welfare, and productivity in sheep. Tooth wear, regardless of its severity, represents a complex process involving the gradual loss of dental hard tissue, which can result in signs and symptoms that may be detrimental to tooth function (Wetselaar *et al.* 2020).

Incisor wear in sheep has been documented primarily in Australia, New Zealand and, to a lesser extent, the UK, with literature from the 1950s–1980s highlighting these regions as the most affected (West 2002; West *et al.* 2018). However, the fact that only a few studies have been conducted elsewhere, makes it a challenge to form a comprehensive global understanding of wear. Observations of permanent incisor wear in sheep in New Zealand were reported as the catalyst for early studies in the late 1950s (Barnicoat 1957, 1959). They were also documented in deciduous incisors of sheep grazing on European pastures in New Zealand (Thurley 1984, 1985). Researchers used the term European pastures to describe high-fertility pastures comprising perennial ryegrass (*Lolium perenne* L) and white clover (*Trifolium repens*) (Barnicoat 1959). Research in the 1960s by Cutress and Healy (1965), Healy and Ludwig (1965) and Healy *et al.* (1967) explored soil ingestion as a potential contributor to incisor wear in sheep. These studies found that supplementary hay or grain feeding helped reduce wear, likely by decreasing reliance on grazing and minimising excessive soil intake, which was particularly elevated in conditions such as heavily stocked or intensively grazed pastures. Soil ingestion is a normal aspect of grazing but can become excessive under certain conditions, such as overgrazed paddocks, bare pastures, or high stocking densities, which increase the likelihood of ingesting abrasive particles. Soil’s contribution to wear is complex, as factors like stocking rates, genetics, and diet also affect incisor wear (Healy & Ludwig 1965; Healy *et al.* 1967). While these early studies did not establish a direct causal link between soil ingestion and wear, they suggested it may exacerbate incisor erosion when combined with other factors.

Literature building on previous research has strengthened the view that both dirt and silica phytoliths in plants have been shown to reduce tooth volume during chewing (Merceron *et al.* 2016; Ramdarshan *et al.* 2016; Ackermans *et al.* 2020). Phytoliths, abundant in grasses, provide structural support to plants and are a major contributor to the attrition observed in the teeth of grazing animals. Sheep grazing in open habitats face additional challenges due to the passive ingestion of windblown sediments, such as dust and grit, with grasses acting as a ‘sediment trap’. Fine dust is suggested to cause uniform wear, while significant grit ingestion may lead to pathological wear over time (Ackermans *et al.* 2020). The cumulative impact of these abrasives combined with plant phytoliths poses a risk to tooth dentine and enamel, potentially resulting in pulp exposure, decay, and tooth loss (Merceron *et al.* 2016; Ramdarshan *et al.* 2016; Ackermans *et al.* 2020). Literature on tooth wear from plant phytoliths and sediments from soil ingestion comprise the bulk of the recent research on sheep incisor wear. However, sheep health literature states that the precise aetiology of incisor tooth wear has yet to be found (West *et al.* 2018; p 215).

This study, the first in a series of studies of incisor disorders in Merino sheep, aimed to provide an insight into how incisor wear may be related to age and physical factors that have implications for sheep welfare. Few studies have explored the epidemiology of incisor wear in sheep. While some have reported weak associations with age (Agostinho *et al.* 2023), such conclusions may be limited by factors such as sample size, age range, or scoring method. In particular, few studies have concurrently examined the relationship between dental wear and BCS, despite the potential welfare implications of reduced oral function on nutritional status. This study aims to expand the evidence base by assessing incisor wear in a large, age-diverse flock of extensively grazed sheep and examining its relationship with age, bodyweight, and condition. In doing so, it addresses a critical gap in the literature and offers new insight into the welfare implications of dental wear in extensively managed flocks.

This study’s first objective was to measure the length of incisors across ewes of various age groups to find the prevalence of incisor wear, length, and loss. The second objective was to explore the relationship between incisor wear, length, and loss with age, body weight, and BCS.

The following hypotheses were tested: (1) that there would be no relationship between ewe age and incisor quality (length, wear or loss); and (2) there would be no relationships between body factors (weight, BCS) and incisor quality.

These hypotheses were framed using a formal null hypothesis approach to facilitate statistical testing of associations within this specific Dohne Merino sheep population.

## Materials and methods

### Study animals and ethical considerations

The research population comprised of 818 Dohne Merino ewes with an age that ranged from 2 to 10 years. Ewes were selected from a 32,000 Dohne Merino flock grazed on a single extensive pastoral sheep production system in southern Australia. The sheep were grazed all year round on extensive native grasslands where the most predominant species is perennial Curly Windmill grass (*Enteropogon acicularis*). Yearly temperatures ranged from 40°C in summer to 5°C in winter with average annual rainfall measuring 400 mm. The Dohne Merino is a dual-purpose, meat-wool sheep bred for the South African grass veld regions by mating South African Mutton Merino rams to South African Merino ewes (Fourie & Heydenrych 1982).

The University of Edinburgh School of Veterinary Medicine Ethical Review Committee approved this study, approval number 82.21.

### Data collection

**Figure 1.**
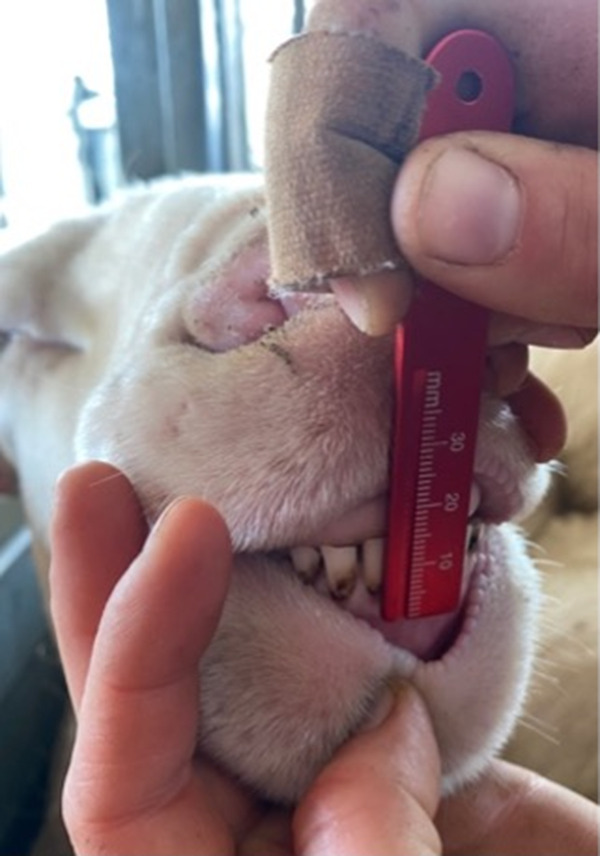
Measurement of the mandibular central incisor (Triadan position 301) in a Merino sheep (*Ovis aries*) using an intraoral endodontic ruler (mm scale). This measurement was used to assess incisor length as part of the evaluation of dental wear. Photograph courtesy of AH.

All study data were collected between November 2021 and February 2022 during regular drenching, vaccination, and other husbandry practices to minimise handling animal welfare risks. While the sheep sampled were those undergoing routine husbandry (e.g. drenching, vaccination), this group was broadly representative of the flock in terms of age and condition. Data collection occurred in a Te-Pari Automatic Weighing Machine (Oamaru, New Zealand) in which an experienced stockperson opened each sheep’s mouth for an oral examination where the incisor assessment was recorded on an odontogram and a single photograph of the incisors was taken with a Canon EO6 90D digital camera (Canon, Japan). Measurements of the two central permanent incisors were taken, with an intra-oral ruler graduated at intervals of 1 mm (Adams Dental Supplies, VIC, Australia). The lower lip was retracted, and the ruler rested against the labial surface of the central incisor. Measurements were made from the free gingiva margin to the highest point of the occlusal tip (Figure 1). The time between restraint, assessment and release was approximately 60 s.

### Bodyweight and body condition assessment protocol

Bodyweight (kg) was obtained using a Te-Pari Automatic Weighing Machine. Ewes were weighed immediately following the dental assessment and BCS assessed using the standard 1–5 scale via palpation of the lumbar spine, following Jefferies (1961).

### Incisor assessment protocol

**Figure 2.**
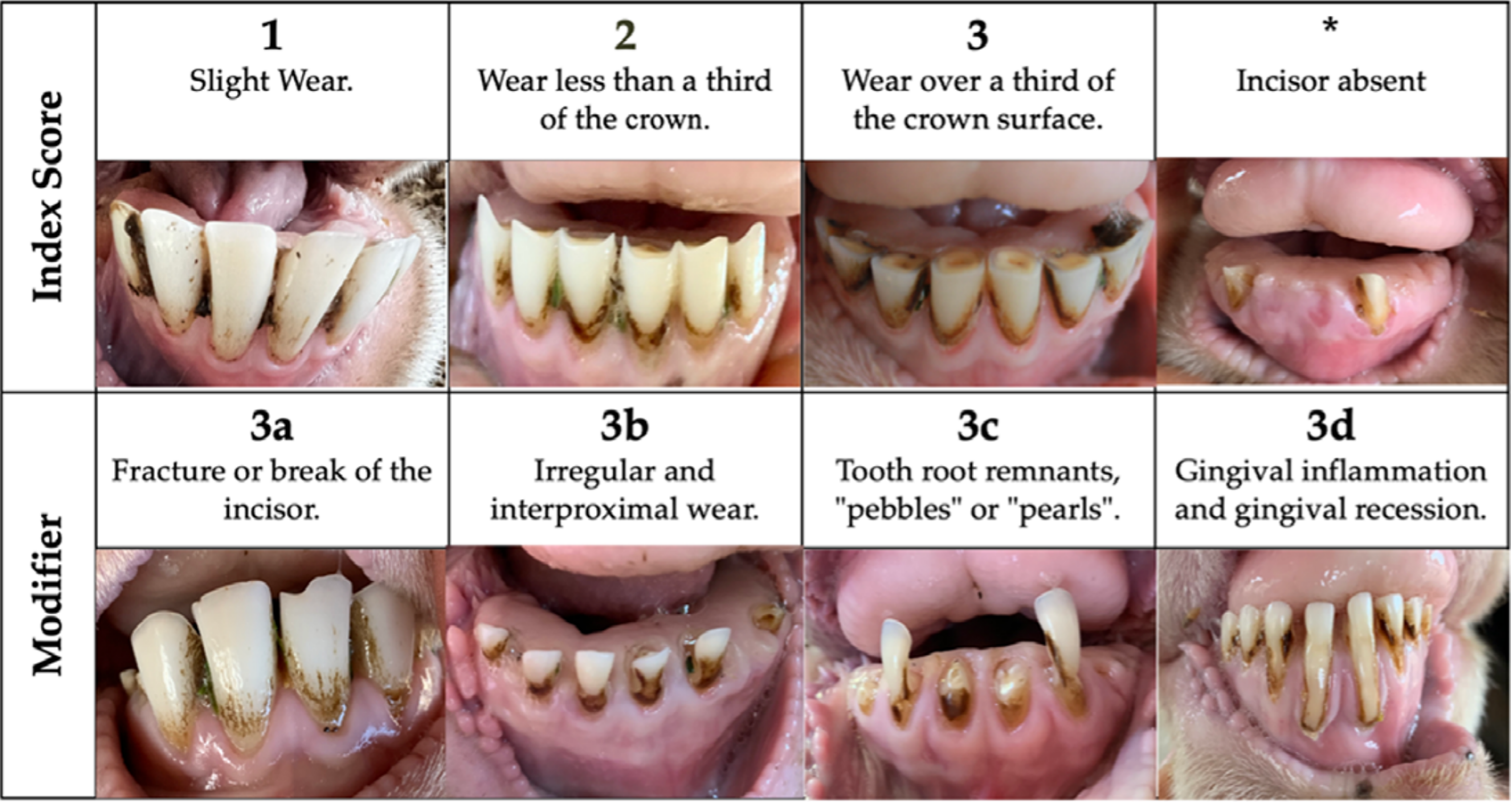
Illustrative Sheep Incisor Assessment Index for evaluating dental wear and pathology in Merino sheep (*Ovis aries*). The index includes: (1) slight wear with smoothing of the occlusal edge; (2) wear affecting less than one-third of the enamel crown with some dentine exposure and cupping; (3) wear extending over more than one-third of the crown surface; (*) absence of the incisor. Modifiers include: (3a) fracture or break of the incisor; (3b) irregular interproximal crown wear; (3c) advanced root exposure with remnant ‘pebble’ or ‘pearl’ formations; (3d) gingival inflammation with pocketing and sulcus recession. All photographs courtesy of AH.

Previous literature provides no established framework for incisor evaluation in sheep, requiring the establishment of an incisor assessment protocol for this study. Incisor assessment was limited to the anatomy visible in live sheep: the incisor crown. The degree of dentine exposure was utilised as the primary measure of tooth wear, utilising the classification parameters developed by Hugoson *et al.* (1988). This assessment used a scoring system where 1 was slight wear, and 3 was wear over one-third of the crown surface, following the scoring system described by Agostinho *et al.* (2023), which was based on the classification parameters of Hugoson *et al.* (1988; Figure 2).

A further modifier score was added to the incisor assessment to assess the presence of other dental disorders, including fractures or breaks in enamel, irregular or interproximal wear of the enamel, tooth remnants or pebbles, and gingival inflammation. While these conditions were not defined as mutually exclusive and could theoretically occur on the same tooth, no instances of multiple conditions co-occurring on a single tooth were observed in this study. The clinical presentation of wear in sheep is not always consistent with shortening due to occlusal surface abrasion. Incisor teeth wear on the interproximal surfaces, eventually reducing them to pebbles (Spence & Aitchison 1986).

Gingivitis was assessed by observing inflammation around the gingival sulcus and lengthening of the incisors’ crowns. Gingival inflammation causes gingival recession, making the incisor crown appear elongated. This apparent lengthening occurs because gingival inflammation leads to gum recession, exposing more of the tooth structure rather than increasing the crown length. Figure 2 (3d) illustrates gingival recession, a key characteristic of periodontal disease progression. While gingivitis and periodontitis are often used interchangeably in sheep literature, they represent different stages of the disease process. Gingivitis is the initial inflammatory response, while periodontitis involves more severe tissue destruction, including periodontal attachment loss. Periodontitis results in the formation of deep pockets around the teeth, which can eventually lead to tooth mobility and loss of incisors (West 2002). Previous studies highlight that incisor loss in sheep is mainly attributed to periodontitis (Spence & Aitchison 1986; Ridler & West 2007). Although dentigerous cysts have been linked to tooth loss in sheep, periodontitis remains the most widely accepted cause of incisor loss (West 2002).

### Data analysis

The collected data from the odontograph were processed in Excel® 2022 (Microsoft Corporation, USA), and statistical analyses were performed using R version 4.2.2 (R Core Team 2022). Due to the lower numbers of animals in the higher age groups, cohorts above seven years were combined for analysis into cohorts 7–10. The 6 year old ewes were unavailable, as they had been moved to another farm. Each of the eight incisors per sheep was assessed individually for wear, resulting in eight separate wear scores. Since each variable was measured once per sheep, no repeated measures or random effects were included in the models. Measurements were taken on the two central incisors (301 and 401) per ewe, the average of the two being used as a continuous outcome. An Excel® MAX(MODE.MULTI) function was used to summarise the data, which returns the highest value of the most frequently occurring wear score across the eight incisors for each sheep. A composite variable, WEARSUM, was created by summing each sheep’s wear scores across all eight incisors. This aggregate measure of wear was then utilised as a continuous predictor in regression models, allowing for a more comprehensive analysis of its potential impact on bodyweight (WGHT) and BCS. To assess the relationship between age (AGE), incisor wear (WEARSUM), incisor length, incisor loss, and their interactions on body weight (WGHT) and BCS, the statistical approaches utilised are summarised in Table 1.

**Table 1.** Summary of statistical models used to evaluate relationships between dental wear, Body Condition Score (BCS), weight, and tooth loss in Dohne Merino ewes (*Ovis aries*; n = 818). Outcome variables, response types, model types, and corresponding R functions are shown

Outcome variable	Type of response	Model used	R function used
Body Condition Score (BCS)	Ordinal	Ordinal Logistic Regression	polr()
BCS (including interaction)	Ordinal	Ordinal Logistic Regression with Age × WEARSUM interaction	polr()
Incisor loss	Count (Poisson)	Poisson Regression	glm(family = poisson)
Bodyweight	Continuous	Linear Regression	lm()
Bodyweight (including interaction)	Continuous	Linear Regression with Age × WEARSUM interaction	lm()
Average incisor length	Continuous	Linear Regression	lm()
Bodyweight and BCS (multivariate)	Continuous & Ordinal	Multivariate Multiple Regression	lm()

Bivariate analyses were conducted using simple linear regression to investigate the individual association between incisor wear and incisor length on bodyweight and BCS. Due to the ordered categorical nature of BCS, ordinal logistic regression was applied to assess its relationship with predictor variables. Given that incisor loss represents a count variable, Poisson regression was used to model its association with age and other predictors. Results were considered statistically significant at *P* ≤ 0.05. Model coefficients, standard errors, and test statistics were extracted using the coef() function, with confidence intervals calculated where relevant. For continuous variables, such as incisor length and bodyweight, linear regression models were employed using the ‘lm()’ function within the ‘stats’ package. Normality, homoscedasticity, and linearity assumptions were checked by visually inspecting residual plots generated with the ‘plot()’ function. The interaction effects between age and incisor wear (AGE × WEARSUM) were examined in both the bodyweight and BCS models to determine whether the impact of wear on these outcomes varied with age.

A multiple linear regression model was used to assess the combined effects of age, BCS, incisor length, and the interaction between age and incisor wear on bodyweight. Incisor loss was initially considered but not retained in the final model due to a lack of significant association with bodyweight. Given the ordinal nature of BCS, ordinal logistic regression was used to evaluate the association between age, incisor wear, and the AGE × WEARSUM interaction on BCS. Since incisor loss is a count variable, Poisson regression was applied to examine its association with age and other predictors. Model fit for ordinal logistic and Poisson regressions were evaluated using McFadden’s R^2^, an appropriate pseudo R^2^ metric for these model types (McFadden 1974). Multicollinearity was assessed using the Variance Inflation Factor (VIF), with all VIF values below three indicating that multicollinearity was not a significant concern.

## Results

**Figure 3.**
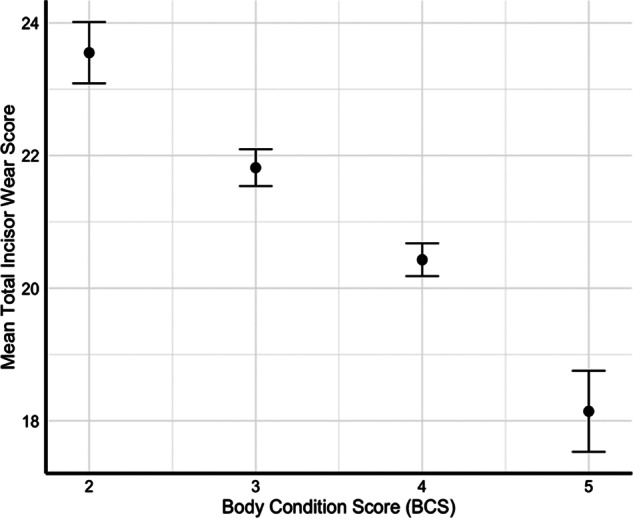
Relationship between Mean Total Incisor Wear Score and Body Condition Score (BCS) in Merino sheep (*Ovis aries*; n = 818). Mean Total Incisor Wear Score decreases as BCS increases, indicating that sheep with higher levels of incisor wear are more likely to exhibit lower body condition. Error bars represent standard error of the mean (SEM). BCS: 1 = emaciated; 5 = obese.

**Figure 4.**
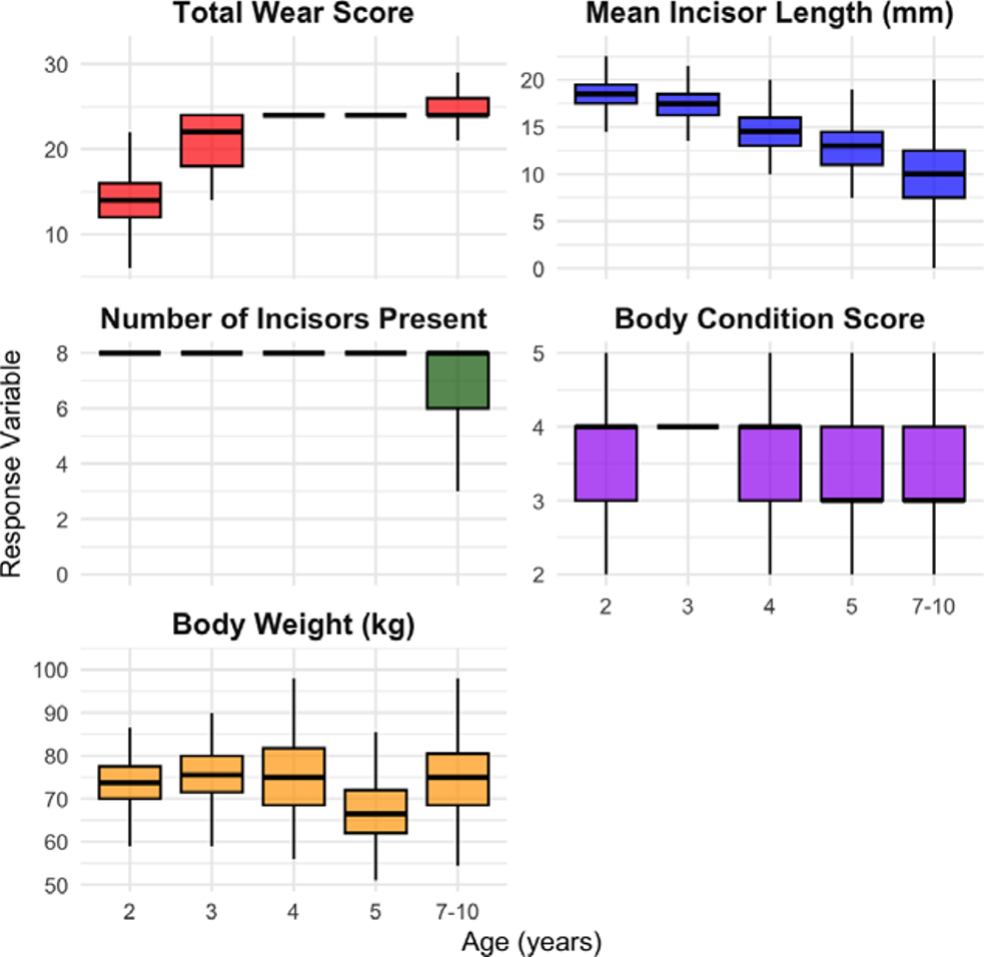
Boxplots showing Total Wear Score, Mean Incisor Length (mm), Number of Incisors Present, Body Condition Score (BCS), and Bodyweight (kg) across five age groups (2, 3, 4, 5, and 7–10 years) in Merino sheep (*Ovis aries*; n = 818). Total Wear Score reflects cumulative tooth wear; incisor length decreases with age, while tooth loss (fewer incisors present) increases in older sheep. BCS (1 = emaciated; 5 = obese) and bodyweight are highest in early adulthood (peaking around 3 years) and decline in older animals.

Table 2 presents summary statistics for bodyweight, BCS, incisor length, and number of incisors present across different age groups. Bodyweight and BCS varied across age groups, with a decline in bodyweight from 76 kg at three years to 67 kg at five years and a bodyweight of around 74 kg in the 7–10 year old cohort. Median BCS decreased from 4 in the 2 year old ewes to 3 in the 7–10 year old cohort. The number of incisors present remained consistent across age groups, with a median of 8. As BCS decreased, the mean total incisor wear score increased, with ewes having a BCS of 2 exhibiting the highest mean wear score (Figure 3). The relationship between age and key response variables, including incisor wear, incisor length, incisor loss, BCS, and bodyweight, are shown in Figure 4.

**Table 2.** Summary of bodyweight, Body Condition Score (BCS), incisor length, and number of incisors present in extensively managed Dohne Merino ewes (*Ovis aries*; n = 818), by age group. Values are shown as mean (± SEM), minimum, maximum, and median (± interquartile range). BCS scored 1–5; Incisor Length (IL) measured in mm; Incisors Present (IP) range 0–8. Incisor loss in 2-year-old group reflects normal shedding of deciduous teeth

Age (yrs)	n	Bodyweight (BW)(kg) Mean (±SEM)	Min BW	Max BW	Body Condition Score (BCS) Median (±IQR)	Min BCS	Max BCS	Incisor Length (IL) (mm) Mean (±SEM)	Min IL	Max IL	Incisors Present (IP) Median (±IQR)	Min IP	Max IP
2	204	73.51 (±0.45)	56.5	92.5	4.0 (±1.0)	2	5	18.55 (±0.12)	13.5	23.0	8 (±0)	6	8
3	163	75.92 (±0.54)	51.0	98.0	4.0 (±0.0)	2	5	17.40 (±0.14)	12.0	23.5	8 (±0)	8	8
4	59	75.92 (±1.27)	56.0	102.5	4.0 (±1.0)	2	5	14.58 (±0.28)	10.0	20.0	8 (±0)	8	8
5	244	66.92 (±0.46)	51.0	94.0	3.0 (±1.0)	2	5	12.86 (±0.16)	0.0	22.0	8 (±0)	4	8
7–10	148	74.40 (±0.71)	54.4	98.0	3.0 (±1.0)	2	5	9.82 (±0.33)	0.0	20.5	8 (±2)	0	8

### Incisor wear and modifiers

The most common type of incisor wear observed was greater than one-third of the crown, particularly in older ewes (Table 3). Ninety-four per cent of the ewes aged 7–10 years exhibited this level of wear, indicating a significant increase in wear with age. In several cases, the wear extended to the point of exposing the pulp chamber. Visible signs consistent with pulpitis, such as darkened or swollen pulp tissue, were also observed in a number of animals, highlighting the potential for pain and infection associated with advanced incisor wear.

**Table 3.** Distribution of the occurrence of incisor wear and modifiers in evaluated Dohne Merino ewes (*Ovis aries*; n = 818) according to age group

Age	Number	Incisor wear n(100%)^1^				Modifier n(100%)^2^			
		1	2	3	*	a	b	c	d
2	204(25)	0(0)	168(82)	36(18)	0(0)	3(1)	21(10)	0(0)	1(1)
3	163(20)	0(0)	51(31)	112(69)	0(0)	6(4)	14(9)	0(0)	23(14)
4	59(7)	0(0)	1(2)	58(98)	0(0)	0(0)	1(2)	0(0)	3(5)
5	244(30)	0(0)	9(4)	233(95)	2(1)	2(1)	23(9)	11(5)	34(14)
7–10	148(18)	0(0)	0(0)	139(94)	9(6)	1(1)	21(14)	52(35)	14(9)
Total	818(100)	0(0)	229(28)	578(71)	11(1)	12(2)	80(10)	63(8)	75(9)

1 Slight wear, 2) Wear < 1/3 Crown, and 3) Wear > 1/3 of the crown and * Missing incisors.

2 The modifier most frequently occurring. a) fracture, b) interproximal wear, c) “pebbles”, and d) gingivitis. Not all sheep had modifiers.

**Figure 5.**
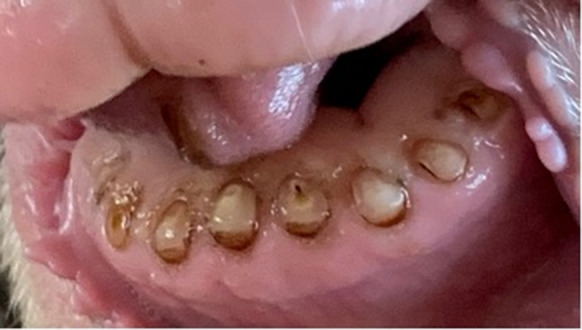
Advanced incisor wear with ‘pebble-like’ tooth remnants in a 10 year old Merino ewe (*Ovis aries*), consistent with severe dental attrition and near-complete crown loss. These rounded root remnants represent an advanced stage of incisor wear identified in the Sheep Incisor Assessment Index. Photograph courtesy of AH.

The assessment of modifiers, fractures, interproximal wear, pebbles, and gingivitis occurred at 11% in the 2 year olds and increased to 59% in the 7–10 year old cohort. Tooth root remnants, called pebbles (Figure 5), were observed in 35% of the 7 to 10 year old cohort, indicating an increase with age. Other modifiers, including fractures or breaks in enamel (modifier a) and irregular enamel wear (modifier b), were less common but still present, further emphasising the increased dental issues in older sheep.

**Figure 6.**
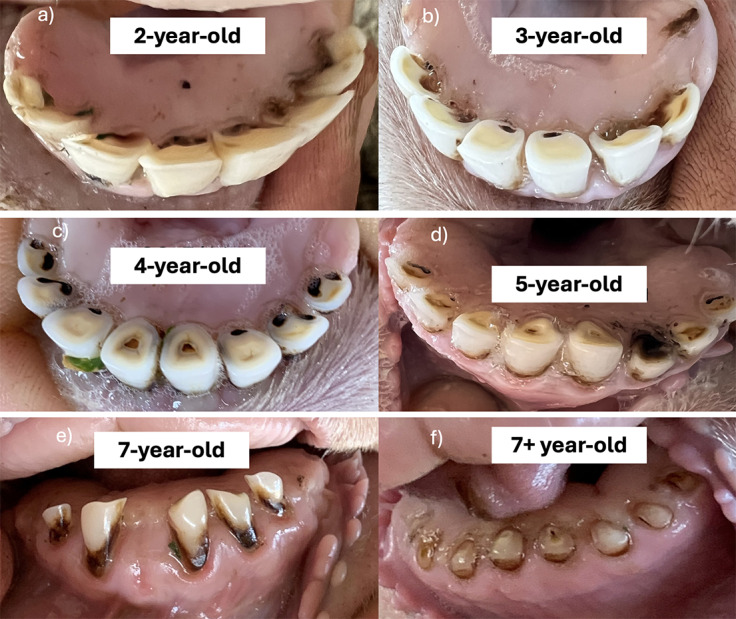
Representative photographs of progressive incisor wear across age groups in Merino sheep (*Ovis aries*; n = 818) that show (a) a 2 year-old with minimal wear, (b) a 3 year old with early crown wear, (c) 4 year old with increased enamel loss and dentine exposure, (d) a 5 year old with more extensive wear and irregular crown surfaces, (e) a 7 year old with advanced wear, crown shortening, and angular deformities and (f) a 7+ year old with near-complete crown loss and root remnants. Photographs illustrate the cumulative nature of dental wear with advancing age and all appear courtesy of AH.

Representative images of the progressive incisor wear observed across age groups are presented in Figure 6, illustrating the increased severity of wear and exposure of dentine with advancing age.

### Regression analysis

Bivariate and multivariable regression analyses were conducted to explore the relationships between incisor wear, age, bodyweight, and BCS. Bivariate models (Table 4) allowed for an initial assessment of individual associations. In contrast, multivariable models (Table 5) accounted for multiple predictors simultaneously to identify the most influential factors on bodyweight and BCS.

**Table 4.** Summary of Bivariate Regression Models used to investigate relationship of incisor wear, length, and loss with age, body weight, and Body Condition Score (BCS) of Dohne Merino ewes (*Ovis aries*; n = 818)

Outcome	Predictor(s)	Model type	R^2^	Significant predictors
Incisor wear	Bodyweight	Linear Regression	0.02	Incisor wear (*P* < 0.001)
Incisor wear	BCS	Ordinal Logistic	0.031^1^	Incisor wear (*P* < 0.001)
Incisor wear	Age	Linear Regression	0.48	Incisor wear (*P* < 0.001)
Incisor length	Bodyweight	Linear Regression	0.02	Incisor length (*P* < 0.001)
Incisor length	BCS	Ordinal Logistic	0.034^1^	Incisor length (*P* < 0.001)
Incisor length	Incisor Wear	Linear Regression	0.40	Incisor length (*P* < 0.001)
Incisor length	Age	Linear Regression	0.60	Incisor length (*P* < 0.001)
Incisor loss	Bodyweight	Poisson Regression	0.000^1^	Not significant (*P* = 0.737)
Incisor loss	BCS	Poisson Regression	0.001^1^	Not significant (*P* = 0.071)
Incisor loss	Age	Poisson Regression	0.003^1^	Incisor loss (P < 0.001)

1 McFadden’s R^2^ values are reported as indicators of model fit for ordinal logistic and Poisson models.

**Table 5.** Summary of multivariable regression models evaluating associations between incisor wear, body weight, Body Condition Score (BCS), incisor length, and age in Dohne Merino sheep (*Ovis aries*; n = 818). Significant predictors, model types, and variance explained (Adjusted R^2^ or McFadden’s R^2^ for ordinal logistic models) are shown

Outcome	Predictor(s)	Model type	Adjusted R^2^ or Model Fit^1^	Significant predictors
Bodyweight	Age, Incisor wear, BCS	Multiple Linear Regression	Adjested R^2^ = 0.302	Age (*P* < 0.001), Incisor Wear (*P* = 0.024), BCS (*P* < 0.001)
Bodyweight	Age × Incisor wear (Interaction)	Multiple Linear Regression	Adjusted R^2^ = 0.302	Interaction (*P* < 0.001)
BCS	Age, Incisor wear, Bodyweight	Ordinal Logistic	R^2^ = 0.238^1^	Age (*P* < 0.001), Bodyweight (*P* < 0.001), Incisor wear (*P* = 0.946 NS)
BCS	Age × Incisor wear (Interaction)	Ordinal Logistic Regression	R^2^ = 0.24^1^	Age (*P* < 0.001), Wear (*P* < 0.001), Bodyweight (*P* < 0.001), Interaction (*P* < 0.001)
Incisor length	Age, Incisor wear, BCS, Bodyweight	Multiple Linear Regression	Adjusted R^2^ = 0.6294	Age (*P* < 0.001), Wear (*P* < 0.001), Bodyweight (*P* = 0.006), BCS Quadratic (*P* = 0.017)

1 McFadden’s R^2^ values are reported as indicators of model fit for ordinal logistic models.

#### Association between incisor wear, age, bodyweight and BCS

Incisor wear showed a significant inverse association with bodyweight (R^2^ = 0.02, *F*_1,816_ = 15.41; *P* < 0.001), where increased incisor wear was associated with lower bodyweights. A similar inverse relationship was observed between incisor wear and BCS (Z = –7.45, Coef = –0.11, SE = 0.014; *P* < 0.001), indicating that increased incisor wear was associated with lower BCS. The model accounted for approximately 3.07% of the variance (see Table 4). Additionally, age was strongly associated with incisor wear (R^2^ = 0.48, *F*_1,816_ = 745.13; *P* < 0.001), indicating that as sheep age, incisor wear becomes more pronounced.

#### Association between incisor length, age, bodyweight and BCS

A significant correlation was found between incisor length and bodyweight (R^2^ = 0.02, *F*₁,₈₁₆ = 18.37; *P* < 0.001), with shorter incisor lengths associated with lower bodyweights. BCS was significantly associated with incisor length (Z = 7.90, Coef = 0.13, SE = 0.017; *P* < 0.001), where lower BCS were linked to shorter incisors, the model accounted for approximately 3.4% of the variance (see Table 4). Further bivariate analysis showed a strong association between incisor length and incisor wear (R^2^ = 0.40, *F*₁,₈₁₆ = 554.3; *P* < 0.001), suggesting that central incisor length could be a reliable indicator of incisor wear. Incisor length was strongly associated with age (R^2^ = 0.598, *F*₁,₈₁₆ = 1,213; *P* < 0.001), with each additional year of age associated with a 1.47-mm reduction in incisor length (Coef = –1.47, SE = 0.042).

#### Incisor loss and relationship with age, bodyweight and BCS

Incisor loss was significantly associated with age (Z = –3.50, Coef = –0.021, SE = 0.0059; *P* < 0.001), suggesting that older sheep lost more incisors. However, age alone accounted for only a small proportion of the variance (see Table 4). No significant association was found between the number of incisors present and bodyweight (Z = 0.34, Coef = 0.0005, SE = 0.0015; *P* = 0.737). Similarly, Poisson regression showed that BCS was not a strong predictor of the number of incisors present (Z = 1.80, Coef = 0.0726, SE = 0.0403; *P* = 0.071), accounting for approximately 1.2% of the variance (see Table 4). These results suggest no clear association between BCS or bodyweight and the number of incisors present.

## Multivariable analysis

Multivariable models were used to explore the combined associations between multiple predictors and outcomes for bodyweight, BCS, and incisor length. A summary of the multivariable models is presented in Table 5.

### Bodyweight

A multiple linear regression model indicated that age, incisor wear, and BCS were significant predictors of bodyweight (Adjusted R^2^ = 0.3021). A multiple linear regression model indicated that age, incisor wear, and BCS were significant predictors of bodyweight (Adjusted R^2^ = 0.3021). Age was positively associated with weight (Coef = 0.59, SE = 0.16, t = 3.68; *P* = 0.0002), while greater incisor wear was associated with lower weight (Coef = –0.15, SE = 0.07, t = –2.26; *P* = 0.024). BCS remained strongly positively associated with weight (Coef = 13.24, SE = 0.81, t = 16.32; *P* < 0.001).

### Body Condition Score (BCS)

A multivariate multiple linear regression model was used to assess the relationship between age and incisor wear, examining both bodyweight and BCS. For BCS, age was significantly associated (Coef = –0.104, SE = 0.0165, t = –6.30; *P* < 0.001), while incisor wear was not significant (Coef = –0.0104, SE = 0.0072; *P* = 0.148). The model explained approximately 11.1% of the variance in BCS (Adjusted R^2^ = 0.1111).

### Incisor length

A multiple linear regression model showed that age, incisor wear, and BCS significantly predicted incisor length (Adjusted R^2^ = 0.6294). Increasing age was strongly associated with shorter incisor length (Coef = –1.204, SE = 0.042; *P* < 0.001), as was increasing wear (Coef = –0.161, SE = 0.041; *P* < 0.001). Bodyweight was positively associated with incisor length (Coef = 0.036, SE = 0.013; *P* = 0.006), while the quadratic term for BCS was also weakly significant (Coef = –0.554, SE = 0.232; *P* = 0.017).

### Model selection and limitations

While incisor wear significantly predicted bodyweight (Coef = –0.225; *P* = 0.005), it did not predict BCS (*P* = 0.148). Similarly, age predicted BCS (Coef = –0.104; *P* < 0.001), but not bodyweight (*P* = 0.948). Given the relatively low variance explained by these models (Adjusted R^2^ = 0.0185 for weight; see Table 5), separate models were used rather than a multivariate multiple regression approach.

Multicollinearity was examined. Age and incisor wear showed a strong correlation (R^2^ = 0.76; *P* < 0.001), which may affect the variance estimates. However, all Variance Inflation Factor (VIF) values remained below accepted thresholds (VIF < 3), suggesting multicollinearity was not a major concern. Given that BCS is an ordinal outcome, it was most appropriately analysed using ordinal logistic regression rather than a linear multivariate model.

### Exclusion of incisor loss

Incisor loss was evaluated but did not significantly predict bodyweight (*P* = 0.237) or BCS (*P* = 0.071) and the model explained little variation in these outcomes (see Table 5). For this reason, incisor loss was excluded from the final multivariable models to preserve parsimony. Additionally, due to a moderate correlation between incisor length and wear (R^2^ = 0.40; *P* < 0.001), and because incisor wear was the primary focus of this study, incisor length was not retained in the final multivariable models to avoid redundancy.

## Discussion

This study is one of the first to assess incisors over differing age groups while simultaneously measuring body condition and weight factors impacting sheep welfare. Given that the literature on incisor wear and loss is somewhat fragmented, often outdated, and frequently based on studies carried out in controlled environments or post mortem, we initially hypothesised that there would be no significant relationships between age, body condition, and incisor wear characteristics. Contrary to our formal null hypothesis, the findings indicate that incisor wear significantly increases with age, with the oldest ewes (7–10 years) exhibiting the greatest wear, including loss of more than one-third of the tooth crown. While Agostinho *et al.* (2023) observed substantial tooth wear in Brazilian sheep, they did not report a statistically significant association between wear and age. Our findings, therefore, contribute new evidence that, in extensive Australian field conditions, age is strongly and positively associated with cumulative incisor wear. Similarly, sheep with shorter incisors exhibited lower BCS, suggesting that incisor wear in sheep is pathological, impairing the ability to access nutrition and maintain body condition.

Previous studies have shown sheep incisors worn down to gum level, producing what has been called *pebbles* in sheep as young as two years old (Spence & Aitchison 1986). In contrast, in our study population, pebbles were first observed in sheep at five years of age, with a prevalence of 5%. By the 7–10 year age group, 35% of sheep exhibited pebble-like remnants (Figure 5), suggesting that these advanced dental pathologies may reflect not only the effects of ageing but also the accumulation of environmental and dietary influences over time. The difference in the age at which advanced wear was observed between our study and previous UK studies may reflect a combination of factors, including breed differences (Dohne Merino vs Scottish Blackface), environmental and climatic conditions, and variation in grazing systems and pasture composition. Differences in tooth eruption timing, forage abrasiveness, and nutritional management may also contribute to population-level differences in the onset and progression of incisor wear.

The presence of pebbles did not correspond with a sharp decline in bodyweight and BCS, indicating that these sheep may have adapted to their dental limitations, possibly by modifying their grazing strategies or forage selection. Additionally, the strategic culling of non-productive sheep as part of targeted farm management may have influenced the study outcomes in ways that could not be directly measured. However, the observation that a significant proportion of older ewes retain incisors with advanced wear highlights the variability in how dental disorders impact individual animals. This suggests that some ewes, particularly those that remain in the flock despite advancing age, may possess certain adaptive traits, allowing them to maintain enough body condition to avoid removal from the flock.

Farm animal welfare cannot be measured directly, but regular assessment of nutritional status remains a cornerstone of effective management. In sheep, variations in BCS can have a significant impact both regarding welfare and productivity. Animals with low BCS are more susceptible to disease, reproductive failure, and poor lamb-rearing outcomes, while those with excessively high BCS face increased risks of metabolic and lambing complications (Plummer *et al.* 2021; Temenos *et al.* 2024). Both extremes compromise individual welfare and threaten the economic sustainability of the flock. Although BCS and bodyweight are often regarded as production traits, they are widely recognised as animal-based welfare indicators in sheep, especially when changes reflect an animal’s ability to maintain nutritional status during physiological challenges, such as dental wear (Munoz *et al.* 2018; Plummer *et al.* 2021).

This study explored the relationship between incisor wear, length and loss with age, body weight, and BCS. Contrary to our second hypothesis, incisor wear showed significant implications for bodyweight and ability to maintain body condition. The decline in BCS with age is consistent with research by Semakula *et al.* (2020) in New Zealand with Romney Ewes and Gonzalez *et al.* (1997) in Uruguay with Merino and Corridale ewes, both of which found a linear relationship between BCS and age. Semakula *et al.* (2020) also found that BCS and live weight in sheep vary linearly and can be predicted using simple linear regression. The significant relationships between age, incisor wear, weight and BCS underscore the complex interaction between ageing and incisor health in sheep.

Incisor wear increased with age, with older sheep exhibiting greater cumulative wear. However, the impact of wear on body condition and weight appeared to be more pronounced when wear occurred at a younger age. Incisor wear was a significant predictor of BCS, with higher wear scores associated with lower BCS. This suggests that as sheep’s incisors wear down, the ability to graze effectively is compromised, leading to reduced fat reserves and poorer body condition. A significant inverse relationship was also observed between body weight and increasing incisor wear, reinforcing that incisor wear limits grazing ability, leading to nutritional deficits and lower body condition over time. These findings have welfare implications, as compromised incisor integrity may contribute to a declining condition in ageing sheep. While the amount of incisor wear increased with age, our interaction models suggest that the impact of wear on production outcomes such as BCS may be more pronounced when wear develops in younger sheep. Early onset wear may therefore have a disproportionately greater welfare and production impact compared to equivalent wear levels occurring later in life. In contrast, the weaker association of wear on BCS in older sheep may reflect selection bias, as only more resilient individuals remain in the flock due to natural culling or management decisions. Further studies are needed to determine whether older sheep develop compensatory grazing strategies or if the observed trend results from the prior removal of individuals most affected by dental wear.

As incisor length decreases with age, there is a corresponding decline in bodyweight and BCS in unadjusted analyses. However, in multivariable models, incisor length was not a significant predictor of BCS, suggesting that the observed relationship may be primarily driven by age rather than a direct effect of incisor length on body condition. This highlights the importance of considering age as a key factor influencing dental wear and overall body condition in ewes. The central incisor length measured in this study was comparable to previous findings from studies in New Zealand. Healy & Ludwig (1965) measured 5 year old sheep and reported a mean incisor length of 3.8 mm in high-wear sheep, 8.9 mm in medium-wear sheep, and 14 mm in low-wear sheep. Cutress *et al.* (1972) reported a mean length of 10 mm in 4 year old sheep, while Suckling and Rudge (1977) measured sheep aged 3 to 9 years and found a mean incisor length of 17.9 mm. Although recent studies have not reported on incisor length, the finding of a mean central incisor length of 10 mm in sheep aged 7–10 years in this study is consistent with historical reports. Ludwig *et al.* (1966) noted an annual incisor length loss of up to 6.35 0mm in a different environment. In contrast, the current study observed a different rate of loss, with a mean incisor length of 19 mm in 2 year old sheep and 10 mm in 7–10 year old sheep, suggesting an average annual loss of about 1.5 mm.

Tooth wear or loss impairs food intake (Hongo *et al.* 2004), potentially leading to persistent hunger, weight loss, and exhaustion, core components of negative welfare states. Further research is needed to understand the broader welfare consequences, particularly in pregnant ewes, where adequate nutrition is essential for foetal development, milk production, and maternal care, however tooth loss is recommended as a welfare indicator in sheep, together with assessment of BCS and analysis of lambing mortality records (Richmond *et al.* 2017). The link between incisor function and body condition also raises concerns about hunger and prolonged foraging efforts, which could alter time budgets and increase competition for resources.

This study found an incisor loss rate of only 1%, much lower than previously reported figures. Despite the low number of incidences, a significant relationship with age was detected. A 1965 study on castrated male Australian Merinos reported that by the age of 7 years, 35% of sheep had retained all their incisors, 51% had missing incisors, and 14% had lost all incisors (Bath *et al.* 1965). Higher rates of incisor loss were noted in Scottish hill sheep, with 60% affected (Aitchison & Spence 1984), and in a New Zealand flock affected by periodontitis, where 48% had incisor loss (Orr & Chalmers 1988). The lower rates of incisor loss were unable to show a relationship between tooth loss and BCS or bodyweight. Dove and Milne (1991) studied 96 lactating ewes but could not determine a definitive impact of missing teeth on milk production. However, they found that reductions in ewe live weight were strongly associated with higher incisor scores, whereas declines in condition scores, though still significant, were less pronounced. There are limited studies on the influence of incisor loss on body condition in sheep; one study was conducted in 1988 in New Zealand, considering three farms with a high prevalence of periodontal disease, with one farm showing a significant association between periodontal disease, with periodontal disease measured as incisor loss, and body condition or weight (Orr & Chalmers 1988). In Australia, Williams (1993) reported that sheep with tooth loss exhibited reduced wool production, with a 2.6% reduction in fleece weight and a 2.3% reduction in bodyweight. The differences observed between our findings and those of previous studies may reflect a combination of factors, including differences in breed susceptibility, climate, soil composition, forage abrasiveness, pasture management, and nutritional regimes across production systems. Variation in scoring methodologies and wear classification systems may also contribute to differences between studies.

As this study was conducted in a single, extensively managed commercial flock, it is possible that some animals with severe dental disorders were indirectly removed through routine culling based on body condition or reproductive performance, rather than formal dental assessment. Dental health is not routinely scored or used as a direct culling criterion in this flock; however, advanced dental disease may contribute to removal through its impact on general health and productivity. This may partially influence the observed prevalence and age distribution of advanced wear compared to other studies.

In the multivariate model, mild incisor loss appeared to have little immediate effect on BCS. However, the non-linear association suggests a tipping point where progressive tooth loss may increasingly limit effective grazing, which contributes to reduced condition and potential welfare compromise. These findings highlight the importance of monitoring dental health as part of ongoing welfare assessment.

### Animal welfare implications

This study highlights the prospective role of dental health, particularly incisor wear and tooth loss, in shaping potential welfare outcomes in sheep. The incisor apparatus is essential for effective grazing and feed intake, and its deterioration through wear or tooth loss can impair an animal’s ability to maintain adequate nutritional status. In this study, increased incisor wear was associated with lower bodyweight, and tooth loss was linked to reduced BCS, highlighting dental health as a relevant factor in welfare assessment. In extensively managed systems, incorporating regular dental assessments alongside BCS could improve the identification of animals at risk of nutritional compromise. As the ability to access nutrition is vital to welfare, especially during physiologically demanding periods such as pregnancy or lactation, incisor health may act as a valuable yet under-utilised welfare indicator. Further research is necessary to investigate the prevalence of these conditions and determine the most effective methods for monitoring and managing their impact across various production systems.

## Conclusion

This study reinforces the value of BCS as both a practical management tool and an indicator of animal welfare in sheep. When interpreted in conjunction with incisor health assessments, including tooth loss, BCS offers meaningful insights into an animal’s ability to meet nutritional demands, particularly in extensive systems where dental wear may go unnoticed.

The associations found in this study between incisor wear, bodyweight, and BCS emphasise the importance of dental integrity in maintaining sheep health and productivity. While previous research has focused on incisor loss, as a diagnosis of periodontitis, our findings suggest that wear and length are more critical welfare indicators. The absence of a significant relationship between incisor loss and production traits challenges long-standing assumptions regarding its role in sheep health. Instead, progressive incisor wear appears to have a more pronounced effect on the ability of sheep to access sufficient nutrition, reinforcing the need for continued monitoring of dental wear as a welfare indicator.

As grazing ruminants, a functional incisor apparatus is essential. A significant inverse relationship between BCS and incisor wear highlights the impact of dental health on overall sheep welfare. Previous studies have not focused on the impact of incisor wear on body condition, highlighting the need for further investigation into how these specific dental issues affect sheep over time. Strong associations between incisor length and wear suggest that average incisor length could be a reliable indicator of wear severity. Multivariate analysis revealed age and bodyweight as significant predictors of BCS, while incisor length and wear had less impact when controlling for these variables. Wear of incisors was also present in younger ages than previously documented, suggesting that deterioration of incisors, even in the early stages, may impair the ability to graze efficiently, which could impact nutrient intake and body condition over time.

While incisor loss was rare in this study (1%), a range of other incisor conditions were observed, with pebbles (8%) and gingivitis (9%) being more frequently recorded than incisor fractures (2%). The high prevalence of pebbles in the 7–10 year age group (35%) suggests that wear may contribute to structural changes in the teeth before complete loss occurs. This supports the hypothesis that incisor wear progresses through multiple stages, potentially leading to functional impairment before outright tooth loss. Furthermore, gingivitis highlights the role of periodontal health in long-term incisor viability. This connection indicates that preventive incisor care, potentially through assessment and better management of grazing conditions and diet, could improve the welfare of older ewes. Our findings highlight that while age and bodyweight are significant predictors of BCS, the expected impact of incisor wear on BCS was more complex than initially hypothesised. These results suggest that the effects of dental wear on BCS may be intertwined with age and weight, requiring further investigation. Notably, our study provides evidence that age is a significant factor in the progression of incisor wear, in contrast to a number of previous studies, which did not observe this association. This finding is crucial for understanding the broader context of sheep welfare, where incisor function is essential for maintaining body condition and weight.
